# The role of feedback for sensorimotor decisions under risk

**DOI:** 10.1167/jov.26.1.13

**Published:** 2026-01-22

**Authors:** Christian Wolf, Artem V. Belopolsky, Markus Lappe

**Affiliations:** 1 Institute for Psychology, University of Münster, Münster, Germany; 2 Department of Experimental and Applied Psychology, Vrije Universiteit Amsterdam, Amsterdam, Netherlands

**Keywords:** saccade eye movements, aiming, movement planning, feedback, reinforcement

## Abstract

For goal-directed movements like throwing darts or shooting a soccer penalty, the optimal location to aim depends on the endpoint variability of an individual. Currently, there is no consensus on whether people can optimize their movement planning based on information about their motor variability. Here, we tested the role of different types of feedback for movement planning under risk. We measured saccades toward a bar that consisted of a reward and a penalty region. Participants either received error-based feedback about their endpoint or reinforcement feedback about the resulting reward. We additionally manipulated the feedback schedule to assess the role of feedback frequency and whether feedback focusses on individual trials or a group of trials. Participants with trial-by-trial reinforcement feedback performed best. They were less loss-aversive, had the least endpoint deviation from optimality, and showed more consistent performance at the group level. This combination of reduced between-participant variability and the improved alignment with optimality suggests that reinforcement feedback about a single movement is particularly effective to optimize movement planning under risk.

## Introduction

Even if we intend to perform the same movement multiple times, the movement and its outcome will not be identical, but they will vary somewhat from one time to the next. Consider, for example, shooting a penalty in soccer. To succeed, the ball must move toward the goal and overcome the goalkeeper. The latter is more likely when the ball is aimed close to the goalpost. But even if we always aim for the lower left corner, our shot will sometimes miss the goal or hit the goalpost instead, whereas in other cases, the ball will land at a more central location of the goal. In goal-directed aiming tasks like shooting a penalty or throwing a dart, the optimal location to aim for is determined by the motor variability of an individual. An experienced soccer player may aim close to the goalpost because they are aware that the ball will land close to the targeted location most of the time. A less experienced player, however, whose shooting is more variable, may repeatedly fail when aiming for the same location, because the ball will frequently hit the goalpost or even land outside of the goal—reducing the chance to score to zero. The inexperienced player, therefore, benefits from aiming at a less extreme location (i.e., closer to the goal center).

Sensorimotor decision tasks like shooting a penalty or throwing darts can be considered a case of movement planning under risk, and performance in these tasks depends on sensory uncertainty, motor uncertainty, and the reward structure of the task (for reviews, see Trommershäuser, Maloney, & Landy, 2008; Wolpert & Landy, 2012). The reward structure refers to the magnitude of reward and penalty, as well as the relative size of the reward and penalty region. Consequently, the reward structure can be manipulated, for example, by changing the relative size of the reward and penalty region or by imposing a different ratio between reward and penalty. Sensory and motor uncertainty, on the other hand, can be considered a characteristic of the participant performing the task. Previous research has shown that movement planning under risk considers both the reward structure of the task and the inherent motor variability (Tatai, Straub, & Rothkopf, 2025; Trommershäuser, Maloney, & Landy, 2003a; Trommershäuser, Maloney, & Landy, 2003b; Zhang, Daw, & Maloney, 2013). Moreover, when feedback about endpoints is perturbed, thereby increasing the inferred motor variability, people adjust their behavior and select a less risk-seeking point to aim (Trommershäuser, Gepshtein, Maloney, Landy, & Banks, 2005). Although movement planning has been shown to maximize the expected gain and is therefore considered close to optimal (Trommershäuser et al., 2003a; Trommershäuser et al., 2003b; Trommershäuser et al., 2005), other studies reported movement planning to be suboptimal (Ota, Shinya, & Kudo, 2016; Ota, Shinya, Maloney, & Kudo, 2019; Wu, Trommershäuser, Maloney, & Landy, 2006). For example, Wu et al. (2006) showed that behavior can be suboptimal with more complex, asymmetric reward structures. But even for simpler reward structures, behavior can be suboptimal, even after prolonged training (Ota et al., 2016) or when additional information is provided to the participant (Ota et al., 2019). To this end, Ota et al. (2019) provided participants with blocked summary feedback after a block of 50 trials. This feedback showed the endpoints of the 50 recent trials, reasoning that observing the distribution of many trials helps to directly infer motor variability. Yet, the additional summary feedback did not improve performance (Ota et al., 2019). However, given that summary feedback was provided incrementally, it is unclear whether blocked summary feedback can improve movement planning or is as effective as trial-by-trial feedback.

In the literature on motor learning and skill acquisition, there is an ongoing debate about the effectiveness of feedback with a reduced frequency compared to trial-by-trial feedback (Fujii, Lulic, & Chen, 2016; Marco-Ahulló et al., 2024; McKay et al., 2022; Ronsse et al., 2011; Salmoni, Schmidt, & Walter, 1984; Schmidt, Young, Swinnen, & Shapiro, 1989; Weir-Mayta, Hamilton, Stockton, & Munoz, 2022; Wulf, Chiviacowsky, Schiller, & Ávila, 2010). According to the guidance hypothesis (Salmoni et al., 1984; Schmidt et al., 1989), trial-by-trial feedback can be superior during skill acquisition but can be detrimental once the feedback is removed, because participants are too reliant upon the presence of feedback. Since then, empirical studies have provided evidence for and against the guidance hypothesis. A recent meta-analysis (McKay et al., 2022) found no clear evidence for the guidance hypothesis and superior retention performance with reduced feedback frequencies. Moreover, trial-by-trial and summary feedback differ in terms of not only feedback frequency but also the focus of feedback: Trial-by-trial feedback provides information about a single movement or its outcome, whereas summary feedback provides information about multiple movements. For movement planning under risk, a focus on multiple movements appears beneficial, because it allows for direct assessment of the motor variability—and thus the crucial property to maximize performance. In contrast, neurocomputational accounts of human motor learning emphasize the evaluation of individual movements and their outcomes (Diedrichsen, White, Newman, & Lally, 2010; Doya, 2000; Feulner, Perich, Miller, Clopath, & Gallego, 2025; Krakauer & Mazzoni, 2011; Shadmehr, Smith, & Krakauer, 2010), suggesting better performance when individual movements can be evaluated.

Motor learning can rely on different processes that use performance-related information in different ways. One prominent distinction emphasizes learning mechanisms that use sensory information about movement errors or outcome or reward information. Sensory error–based processes update motor commands based on the discrepancy between predicted and observed movement consequences, enabling direction-specific corrections (Shadmehr et al., 2010). Outcome-based processes adjust future actions according to their success or failure, without requiring information about the error direction (Doya, 2000; Izawa & Shadmehr, 2011). Although outcome-based updates are often discussed within the broader framework of reinforcement learning, they differ from sensory error–driven mechanisms in the nature of the information they use. These learning principles motivate an important question for movement planning under risk: Does the type of information provided as feedback—sensory error versus reward outcome—shape how people choose an aim point when the optimal solution depends on their own motor variability?

In the present study, we rigorously tested the role of feedback for movement planning under risk using saccade eye movements—a response system with established influences of reinforcement and error-based learning (Madelain, Paeye, & Darcheville, 2011; Pélisson, Alahyane, Panouillères, & Tilikete, 2010). However, our goal was not to distinguish between reinforcement learning and sensory error–based mechanisms. Rather, we use these concepts as a conceptual motivation for why feedback highlighting individual movement outcomes (reinforcement feedback) might influence planning differently from feedback that conveys sensory error information (error-based feedback). In our experiment, we asked participants to make saccades to an elongated bar divided into a reward region and a penalty region. Both reward and penalty increased toward the center of the bar. Thereby, aiming into the rewarded region close to the center of the bar yielded a high amount of reward—yet at the risk of encountering a penalty. We systematically manipulated the feedback modality (error-based feedback, reinforcement feedback), as well as the feedback schedule (trial-by-trial feedback, blocked summary feedback, rolling summary feedback), in a fully crossed between-participant design. Participants received feedback about the obtained score (reinforcement feedback) or about their endpoints (error-based feedback). Moreover, participants received feedback on a trial-by-trial level or feedback about the recent 30 trials (summary feedback). Two groups of participants received summary feedback every 30 trials (blocked summary), whereas two other groups received summary feedback after every trial (rolling summary). A comparison between the blocked summary and rolling summary groups reveals the contribution of feedback frequency, and the comparison between trial-by-trial and rolling summary feedback reveals the contribution of feedback focus—thus, whether feedback focuses on the outcome of individual movements or on a set of movements.

## Methods

### Participants

We recorded data of 120 participants (100 females, 20 males; age range: 18–46; median age: 21). Participants were Psychology students from the University of Muenster and were reimbursed with course credit or with 8€/h. Additionally, participants received a performance-contingent reward that depended on their performance in the experiment. The performance-contingent reward varied between 0€ and 2.40€ (median: 1.90€). Participants had normal or corrected-to-normal vision. The experiment was approved by the Ethics Committee of the Faculty of Psychology and Sports Science at the University of Münster, and participants provided written informed consent before taking part in the experiment.

### Setup and stimuli

Stimuli were presented on an Eizo FlexScan 22-inch CRT monitor (Eizo, Hakusan, Japan) with a resolution of 1,152 × 870 pixels, an effective display size of 40.7 × 30.5 cm, and a refresh rate of 75 Hz. A chin–forhead rest was used to restrict head movements and ensure a viewing distance of 67 cm. Stimulus presentation was controlled via the Psychtoolbox (Brainard, 1997; Kleiner, Brainard, & Pelli, 2007) in MATLAB (The MathWorks, Natick, MA, USA). Eye position of the right eye was recorded at 1000 Hz using the EyeLink 1000 (SR Research, Mississauga, ON, Canada) and the EyeLink Toolbox (Cornelissen, Peters, & Palmer, 2002). All stimuli were presented on a gray background (9.06 cd/m^2^). The EyeLink was calibrated at the beginning and in the middle of each block using a 9-point calibration protocol.

**Figure 1.**
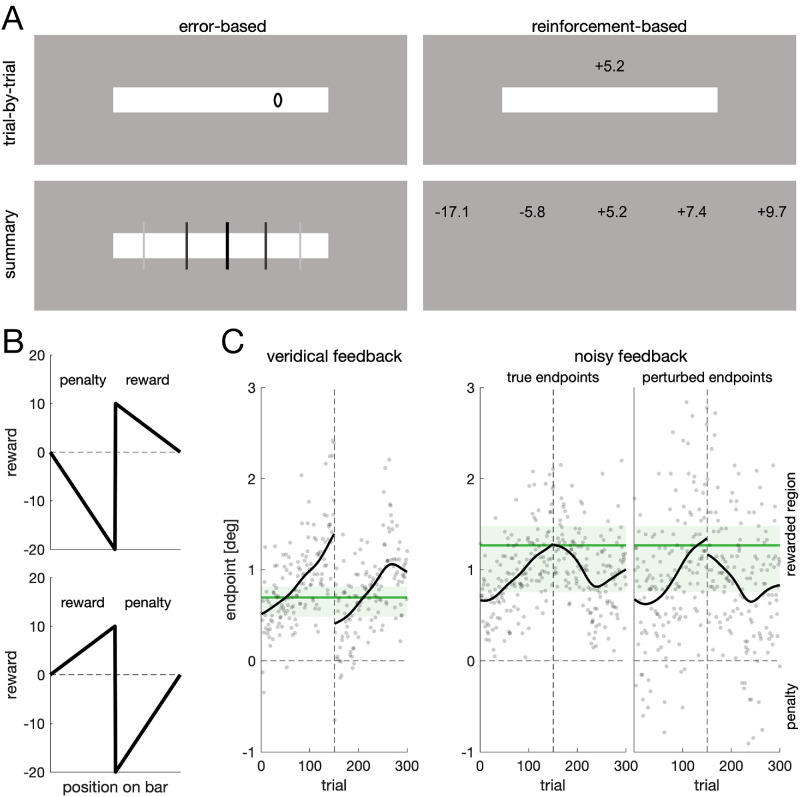
**Design and critical manipulations.** (**A**) Participants were rewarded/penalized for making vertical eye movements to a horizontally elongated white bar. We recorded six groups that received different forms of feedback. Participants received feedback about their endpoint (error-based, left column) or about their obtained score (reinforcement, right column). Participants in the trial-by-trial groups (top row) received feedback about their performance in the recent trial, whereas participants in the summary groups (bottom row) received feedback about their performance in the recent 30 trials. Participants in the blocked summary groups received feedback every 30 trials, and participants in the rolling summary group received feedback after every trial. Summary feedback consisted of the mean, the minimum and maximum, and the 25th and 75th percentiles in terms of position or obtained score. (**B**) Reward structure. Half of the bar was associated with a reward, the other half with a penalty. Importantly, reward and penalty increased toward the bar center. For half of participants, the rewarded region was the right-hand part of the bar. (**C**) Individual data of one participant. Every participant completed two blocks, each with a break after 150 trials (dashed vertical line). In the first block, participants received feedback about their true endpoints (veridical feedback, left). In the second block (noisy feedback, center and right), position noise was added to the provided feedback. Every data point is the endpoint of one trial. Solid black lines are a moving average (σ = 20 trials). Green lines are optimal aim points derived from the variability relative to the moving average. The green shaded area denotes the region of optimality for that individual (Supplementary Figure S1). For noisy feedback, the perturbed endpoints (right) and not the true endpoints (center) were used to compute the optimal aim point.

The stimulus was a horizontally elongated white bar (83.6 cd/m^2^) with a width of 8.1° and a height of 1.04°. It was presented at the horizontal monitor midline, at a vertical eccentricity of either –7.5° or +7.5°. The bar had no sharp visible edges but slowly faded into the background: The outermost 0.15° of each edge was a linear transition into the background. We used a combination of a fixation cross and a bull's eye as a fixation cross (Thaler, Schütz, Goodale, & Gegenfurtner, 2013). To provide feedback in the error-based conditions, we used an ellipsoid with a diameter of 0.3 (horizontal) and 0.5 (vertical) for trial-by-trial feedback and black vertical lines of 1.65° length for summary feedback (Figure 1A). For the reinforcement conditions, we used numbers rounded to one decimal displayed 1.25° above the bar.

### Design and procedure

Participants were instructed to make vertical saccades to a horizontally elongated bar. Half of the bar was associated with a reward, whereas the other half was associated with a penalty. Critically, reward and penalty increased toward the center of the bar, with the penalty being twice as high as the reward (Figure 1B). This information was made explicit to participants. The vertical position of the bar and the reward orientation on the bar were balanced across participants. The asymmetric reward structure ensured that the expectedly few penalties contributed to the overall performance. We recorded six groups that differed in terms of the feedback provided to them. Participants received feedback about their endpoint on the bar (error-based) or about the obtained reward (reinforcement). Moreover, participants received feedback about the recent trial (trial-by-trial) or about the recent 30 trials (blocked summary and rolling summary). Participants in the blocked summary groups received feedback every 30 trials, whereas participants in the rolling summary groups received feedback after every trial. During the first 29 trials, participants in the rolling summary conditions received feedback about all previous trials.

Every participant completed two blocks. In the first block, participants received feedback about their true endpoints (veridical feedback). In the second block (noisy feedback), endpoints (and thus scores) were perturbed by adding position noise (σ = 0.5°) to the true endpoint. Thus, the experiment constituted a 2 × 2 × 3 design, with the within-participant factor *feedback veridicality* (veridical, noisy) and the between-participant factors *feedback modality* (error-based, reinforcement) and *feedback schedule* (trial-by-trial, blocked summary, rolling summary).

At the beginning of each trial, a fixation cross was displayed at the screen center. A trial was started if gaze was within a square region of 2.5° width around the fixation cross for 250 consecutive samples. After a uniform random interval between 200 and 500 ms, the bar appeared. Participants were instructed to quickly look at the bar after its appearance with a single eye movement. A sample was labeled on-target if it was less than 0.5° away from the edge of the bar. The target was classified as selected if gaze was on the bar for 200 samples. The mean gaze position of the last 10 samples was used to provide feedback. In the trial-by-trial conditions, feedback was displayed for 306 ms. Summary feedback was displayed until participants pressed any button on a keyboard. If the target was not selected within 1,200 ms, the target was removed, and the next trial began. Summary feedback was a graphical representation (Figure 1A) of the mean, the minimum and maximum, and the 25th and 75th percentiles in terms of position or score. Participants in the summary groups were told that feedback provided information about the recent 30 trials, displaying the mean (central line or value; see Figure 1A), the values covering the central half of the responses (neighboring elements), and the minimum and maximum (outmost elements).

Every participant completed a demo version of the experiment before proceeding to the main experiment to get familiar with the task. The demo consisted of five trials, and no feedback was provided. Unlike the main experiment, the penalized region of the bar was displayed in dark gray. Both blocks of the main experiment consisted of 300 trials and included a break after 150 trials. Participants were told that their task was the same in the two blocks, yet a different algorithm was used to compute where they had looked. After completing the second block, score points were converted into a monetary reward, with 1,000 points yielding 1€. For the noisy feedback block, the payment was based on the true score or on the perturbed score, depending on what was higher.

### Data analysis

We recorded gaze position of the right eye at 1000 Hz. Saccade onsets and offsets were defined using the EyeLink 1000 algorithm. In the offline analysis, gaze position at saccade offset was used as a saccade endpoint. These offline estimates of saccade endpoints and the online estimates of gaze position (which were used for feedback) were highly correlated, *r* = 0.96, *p* < 0.001. The analysis is based on the offline results. We discarded 21 trials (out of 72,000) because no gaze position on the target was detected.

**Figure 2.**
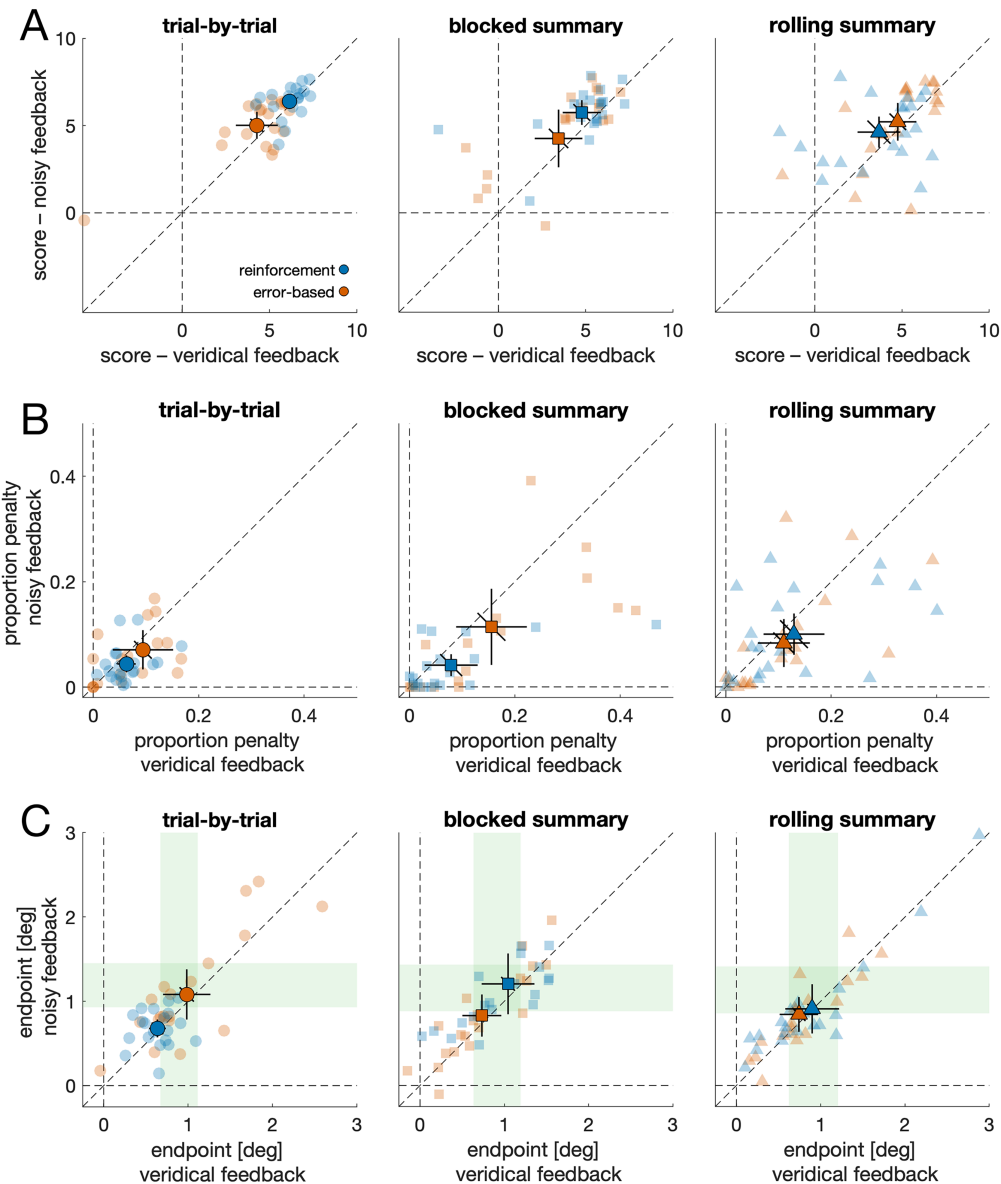
**Performance.** (**A**) Average score per trial for every participant. Every participant first completed a veridical feedback condition before proceeding to the noisy feedback condition. The score in the noisy feedback condition shows the true score, not the perturbed one used for feedback. Each faint data point is the mean of one individual. Solid data points are the group mean. Error bars represent the 95% confidence interval of between-participant variability. Diagonal error bars must be compared to the identity line. (**B**) Proportion of penalized trials for every participant. (**C**) Average endpoint of every participant. Positive values denote average endpoints on the rewarded region; negative values indicate the penalized region. Green regions denote the region of optimality.

The optimal aiming point depends on the motor variability. Under the assumption of a stable aim point across trials, the motor variability is identical to the variability of endpoints. An optimal aim point can then be derived by shifting the entire endpoint distribution to different mean values and computing the resulting overall score for that mean endpoint. The mean endpoint with the highest score can be used as an estimate of the optimal aiming point. However, such an analysis would neglect that participants may change their aiming location over the course of the experiment, either by slow, gradual changes or by sudden changes in strategy. Thus, the observed variability may reflect not only motor variability but also aiming variability (i.e., variability due to aiming at different locations). In consequence, this approach would overestimate the individual motor variability. Alternatively, one may compute the endpoint residuals relative to a moving average (Figure 1C). This way, changes in the aiming location over time can be accounted for. However, the moving average and the resulting residuals will depend on the size of the sliding window that is used to compute the moving average. A common choice is to use a Gaussian weighting function as a sliding window, where the standard deviation of the Gaussian controls the effective width of the window. A small sliding window may result in a noisy moving average that underestimates motor variability (Supplementary Figure S1). A large sliding window, on the other hand, will overestimate motor variability, given that the moving average will approach the individual mean with increasing window size (Supplementary Figure S2). Considering all these limitations, we decided not to provide point estimates of motor variability and thus of optimal performance, but to compute an upper limit (spread around the individual mean) and a lower limit of motor variability (spread around the moving average with the smallest sliding window). We refer to the region between the upper and lower limits as the region of optimality (Supplementary Figure S1). Specifically, to compute the lower limit of motor variability, we used a sliding Gaussian window with a standard deviation of 1 trial. Thus, each trial's moving average was effectively influenced by the trial itself and its immediate neighbors. For Figures 2^3^–4, the depicted regions of optimality indicate the distance from the lower limit (minus 1.96 times the standard error) to the upper limit (plus 1.96 times the standard error) for each participant group. We excluded one participant from the computation of the region of optimality, because this participant alternated between looking at the left and right edges of the bar during the first block, thereby producing unrealistically high values of variability.

**Figure 3.**
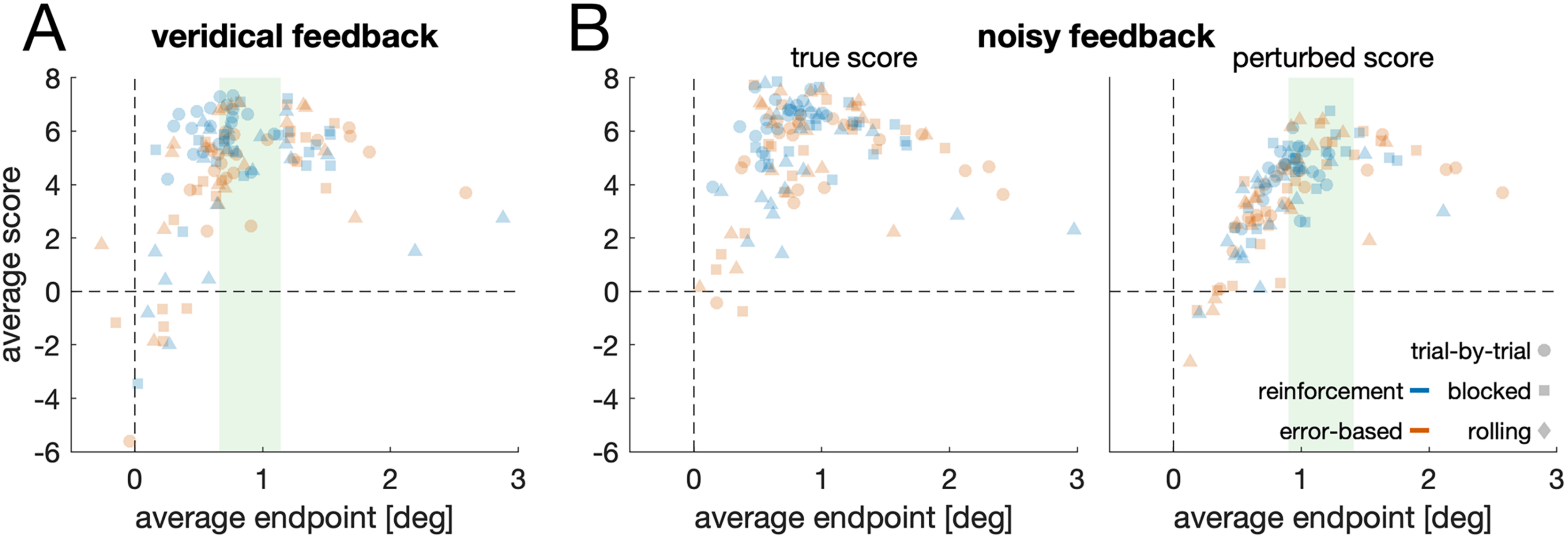
**Scores as a function of endpoints.** Average score as a function of the average endpoint for (**A**) the veridical feedback condition and (**B**) the noisy feedback condition. Each data point is one individual. Green regions denote the regions of optimality.

**Figure 4.**
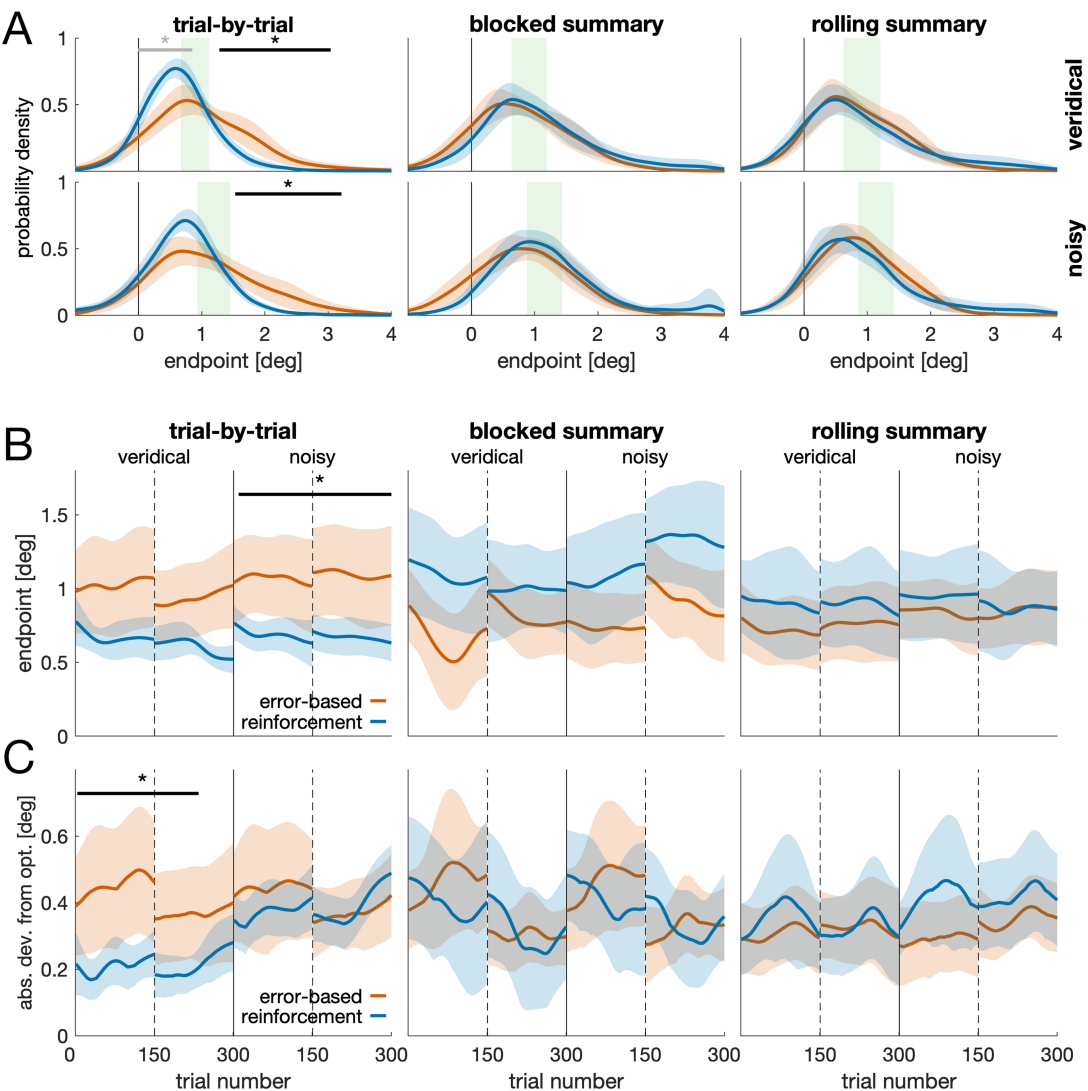
**Endpoints in space and time.** (**A**) Kernel density estimates of saccade endpoints. Horizontal lines and asterisks denote a difference between error-based feedback (orange) and reinforcement feedback (blue), as revealed by a cluster permutation test. Black colors indicate the strongest cluster. (**B**) Endpoints over time for error-based (orange) and reinforcement feedback (blue), separate for the trial-by-trial (left), blocked summary (center), and rolling summary condition (right). Each panel shows data from the veridical feedback condition (left of solid vertical line) and the noisy feedback condition (right of solid vertical line). Dashed vertical lines indicate the break during each experimental block. Solid horizontal lines and asterisks indicate a significant difference between time courses, as revealed by a cluster permutation test. (**C**) Absolute deviation from the region of optimality. Same conventions as in (**B**).

We compared scores and endpoints using a 2 × 2 × 3 analysis of variance (ANOVA) with the within-participant factor *feedback veridicality* (veridical, noisy) and the two between-participant factors *feedback modality* (error-based, reinforcement) and *feedback schedule* (trial-by-trial, blocked summary, rolling summary). The direction of effects was tested using *t*-tests. Inferential statistics are supplemented with effect size estimates, Cohen's *d* for *t*-tests, and partial eta squared, $\eta_{p}^{2}$, for ANOVA results. Postpenalty behavior was analyzed using a 2 × 3 × 5 ANOVA on the saccade endpoints with the between-participant factors *feedback modality* and *feedback schedule* and the within-participant factor *postpenalty trial* (T1–T5; referring to the first through fifth trials following a penalty). To assess the effect of blocked summary feedback, we analyzed data from the blocked summary groups using a 2 × 2 × 2 × 5 ANOVA on the saccade endpoints, with the between-participant factor *feedback modality* and the within-participant factors *feedback veridicality*, *feedback phase* (prefeedback, postfeedback), and *trial lag* (T1 to T5; distance in trials from the feedback event, where negative lags correspond to prefeedback trials and positive lags to postfeedback trials). The results of all ANOVAs are presented in Supplementary Tables S1–S4.

We used cluster permutation tests with 1,000 permutations to compare continuous data (i.e., endpoints over time and kernel density estimates) of two conditions. For each permutation, the assignment of participants to the groups was randomly permuted.

## Results

### Best performance with trial-by-trial reinforcement feedback

To quantify performance, we computed the mean score per trial. Figure 2A shows scatterplots of the noisy score (i.e., score in the noisy feedback condition) over the veridical score (i.e., score in the veridical condition) for the different groups. To compute the noisy score, we used the true score associated with the real endpoints and not the perturbed score that was used for feedback.

Scores were compared with a 2 × 2 × 3 ANOVA. Supplementary Table S1 provides coefficients of all main effects and interactions. On average, scores were higher with reinforcement feedback compared to error-based feedback (main effect feedback modality), *F*(1, 114) = 4.01, *p* = 0.048, $\eta_{p}^{2}$ = 0.034. Crucially, the effect of feedback modality was further modulated by feedback schedule (feedback modality × feedback schedule interaction), *F*(2, 114) = 4.63, *p* = 0.012, $\eta_{p}^{2}$ = 0.075. Although reinforcement feedback was superior to error-based feedback when provided on a trial-by-trial basis (veridical: *t*(38) = 3.08, *p* = 0.004, *d* = 0.97; noisy: *t*(38) = 3.22, *p* = 0.003, *d* = 1.02), we observed no statistically reliable difference with blocked summary feedback (veridical: *t*(38) = 1.60, *p* = 0.119, *d* = 0.50; noisy: *t*(38) = 1.73, *p* = 0.092, *d* = 0.55) or with rolling summary feedback (veridical: *t*(38) = −1.39, *p* = 0.174, *d* = −0.44; noisy: *t*(38) = −0.89, *p* = 0.382, *d* = −0.28). Moreover, for reinforcement feedback, scores were higher for trial-by-trial feedback compared to rolling summary feedback (veridical: *t*(38) = 3.97, *p* < 0.001, *d* = 1.26; noisy: *t*(38) = 3.70, *p* < 0.001, *d* = 1.17), as well as compared to blocked summary feedback, but only in the veridical condition (veridical: *t*(38) = 2.43, *p* = 0.0198, *d* = 0.77; noisy: *t*(38) = 1.58, *p* = 0.122, *d* = 0.50). The interaction between feedback modality and feedback schedule was primarily driven by the rolling summary groups, which showed a tendency toward the opposite pattern compared to the trial-by-trial and blocked summary conditions (Figure 2A). In contrast, the trial-by-trial and blocked summary conditions exhibited similar mean differences between feedback modalities, although the latter appeared to be influenced by a few outliers rather than a consistent group-level effect.

Additionally, the ANOVA revealed a main effect of feedback veridicality, reflecting that noisy scores were higher than veridical scores, *F*(1, 114) = 13.694, *p* < 0.001, $\eta_{p}^{2}$ = 0.107. This is expected if, first, our manipulation of feedback veridicality was successful (i.e., people became more cautious with noisy feedback) and, second, participants lost the most points in the veridical condition by being too risky.

Performance in the task depends on aiming at a rewarded location close to the center of the bar while minimizing the number of penalties. A low score can result from being too cautious (low reward, few if any penalties) or from being too risky (high reward, but also a high number of penalties). Indeed, over all groups, the mean score was strongly correlated with the proportion of trials in the penalty region, *r*(238) = −0.85, *p* < 0.001. Descriptively, the proportion of penalties was lowest with reinforcement feedback, in both the trial-by-trial and blocked summary groups (Figure 2B). Yet, the ANOVA only yielded a main effect of feedback veridicality, *F*(1, 114) = 14.89, *p* < 0.001, $\eta_{p}^{2}$ = 0.116, reflecting a lower number of penalties in the noisy condition (unperturbed data).

### Trial-by-trial reinforcement feedback enables high reward endpoints

The relationship between the individual mean endpoint on the bar and the mean score per trial was inverted U-shaped (Figure 3), with a peak at approximately 1° (veridical feedback condition): For endpoints above 1°, the score was lower the further the mean endpoint was away from the bar center, *r*(34) = −0.75, *p* < 0.001, whereas the opposite was true for endpoints below 1°, *r*(82) = 0.67, *p* < 0.001. This turning point of approximately 1° coincides with the inferred region of optimality: In the veridical condition, the lower limit of optimality was *M_lower_* = 0.69° (*SD_lower_* = 0.14°), and the mean upper limit was *M_upper_* = 1.10° (*SD_upper_* = 0.24°). In the noisy condition, however, the lower limit was *M_lower_* = 0.93° (*SD_lower_* = 0.12°), and the upper limit was *M_upper_* = 1.38° (*SD_upper_* = 0.18°). Thus, across the whole sample, we obtained a region of optimality that varied between 0.66° and 1.14° for the veridical condition and between 0.90° and 1.41° for the noisy condition. Please note that the regions of optimality are not identical for the different groups (Figures 2C and 4A).

Although mean endpoints showed substantial interindividual differences in most of the groups (Figure 2C), participants in the trial-by-trial reinforcement feedback group were most consistent (lowest between-participant variability; Figure 2C), and their mean endpoints were within the region of optimality or risk-seeking (i.e., in between the region of optimality and the bar center; Figure 4A). An ANOVA on the endpoints (Supplementary Table S2) revealed an interaction between feedback modality and feedback schedule, *F*(1, 114) = 4.70, *p* = 0.011, $\eta_{p}^{2}$ = 0.076. For trial-by-trial feedback, endpoints were lower (i.e., closer to the bar center) for the reinforcement versus the error-based condition (veridical: *t*(38) = 2.44, *p* = 0.020, *d* = 0.77; noisy: *t*(38) = 2.67, *p* = 0.011, *d* = 0.84). This was neither the case for blocked summary feedback (veridical: *t*(38) = −1.67, *p* = 0.103, *d* = −0.53; noisy: *t*(38) = −1.79, *p* = 0.008, *d* = −0.57) nor for the rolling summary (veridical: *t*(38) = −0.84, *p* = 0.406, *d* = −0.27; noisy: *t*(38) = −0.38, *p* = 0.701, *d* = −0.12). For reinforcement feedback, endpoints were closer to the bar center for trial-by-trial compared to blocked summary feedback (veridical: *t*(38) = 2.59, *p* = 0.013, *d* = 0.82; noisy: *t*(38) = 2.94, *p* = 0.006, *d* = 0.93) but not compared to rolling summary (veridical: *t*(38) = 1.64, *p* = 0.109, *d* = 0.52; noisy: *t*(38) = 1.56, *p* = 0.127, *d* = 0.49).

Moreover, the ANOVA revealed a main effect of feedback veridicality, *F*(1, 114) = 9.24, *p* = 0.003, $\eta_{p}^{2}$ = 0.075, reflecting that endpoints in the noisy feedback condition, *M_noisy_* = 0.93°, *SD_noisy_* = 0.58°, were on average further away from the bar center than in the veridical feedback condition, *M_veridical_* = 0.84°, *SD_veridical_* = 0.56°, *t*(119) = 3.07, *p* = 0.003, *d* = 0.28. This reflects that the noisy feedback manipulation was successful and that participants adjusted their endpoints to become more cautious. Yet, this average shift (Δ*M* = 0.09°) was less than what would be required to maintain a good level of performance, given that the differences in the lower limit (Δ*M* = 0.24) and upper limit of the optimal endpoint (Δ*M* = 0.28) were approximately three times as high.

How can people's suboptimality be characterized? Based on the individual region of optimality, we computed the proportion of rewarded but suboptimal trials and classified them as either risk-seeking (i.e., in between the bar center and the region of optimality) or as cautious/loss-aversive (i.e., beyond the region of optimality). For five of six groups, the fraction of risk-seeking and loss-aversive trials was approximately the same (Figure 4A; Supplementary Table S5), with trial-by-trial reinforcement feedback being the only exception. With veridical feedback, participants in the trial-by-trial reinforcement group had a higher proportion of risk-seeking trials, *M_risk_* = 46.7% [42.8%, 50.6%], and a lower number of cautious/loss-aversive trials, *M_cautious_* = 22.7% [17.0%, 28.4%], compared to the remaining sample, *M_risk_* = 30.1% [26.9%, 33.2%] and *M_cautious_* = 35.5% [30.3%, 40.8%], respectively. This was also true for noisy feedback: *M_risk_* = 58.9% [53.8%, 64.1%] and *M_cautious_* = 12.9% [9.0%, 16.8%] for trial-by-trial reinforcement feedback compared to *M_risk_* = 41.1% [36.7%, 45.5%] and *M_cautious_* = 27.8% [22.6%, 33.3%] for the remaining sample.

Although the ANOVA identifies overall differences between feedback conditions, any systematic temporal structure in the endpoints may violate its assumption of independence. To ensure that our conclusions do not rely on this assumption, we therefore complemented the ANOVA with cluster permutation tests, which allow distributional (Figure 4A) and temporal (Figure 4B) comparisons without requiring independence. First, we estimated each participant's endpoint distribution using a kernel smoothing function and compared distributions of different groups using cluster permutation tests (Figure 4A). For trial-by-trial feedback, we observed a difference between reinforcement and error-based feedback (veridical: *t_sum_* = 475.84, *t_crit_* = 200.35, *p* = 0.001, cluster position: 1.28°–3.04°; noisy: *t_sum_* = 428.54, *t_crit_* = 232.95, *p* = 0.008, cluster position: 1.53°–3.21°).

How much do endpoints deviate from optimality? Based on the time course of endpoints (Figure 4B), we computed the distance of each endpoint to the region of optimality of an individual. Figure 4C shows a moving average of this deviation. The deviation to optimality was lowest with trial-by-trial reinforcement feedback in the veridical condition. A cluster permutation test revealed a difference between reinforcement and error-based feedback, *t_sum_* = 560.9, *t_crit_* = 261.9, *p* = 0.003, trials: 4–234. Importantly, the early onset of this cluster is attributable to the temporal filtering applied to the data. When unfiltered data are used, the difference between conditions emerges later in the experiment.

### Trial-by-trial feedback enables immediate error correction

**Figure 5.**
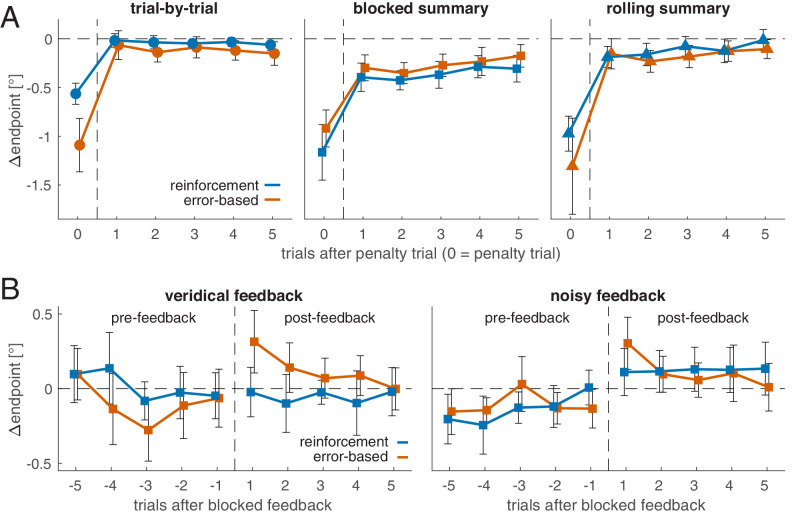
**Posterror and postfeedback behavior.** (**A**) Endpoints following a saccade on the penalized region for trial-by-trial feedback (left), blocked summary feedback (central), and rolling summary feedback (right). The penalty trial itself is shown at position 0 on the x-axis. Endpoints are shown relative to the individual mean (horizontal dashed line). The results in (**A**) and the accompanying analysis combine data from the veridical and noisy feedback conditions. The pattern and conclusions do not change if the analysis is restricted to data from the veridical block. (**B**) Endpoints before and after feedback in the blocked summary condition. Endpoints are shown relative to the individual mean. All error bars are 95% confidence intervals of between-participant variability.

How does movement planning change after making an error? Figure 5A shows endpoints (relative to the individual mean) of the five trials (T1 to T5) following a saccade into the penalty region (with T0 being the penalty trial). Although endpoints immediately following a penalty are not different from the individual mean with trial-by-trial feedback, *M_T1_* = −0.04°, *t*(36) = 0.98, *p* = 0.332, *d* = 0.16, endpoints immediately following an error remain below the individual mean, for both blocked summary feedback, *M_T1_* = −0.34, *t*(38) = 6.90, *p* < 0.001, *d* = 1.10, and for rolling summary feedback, *M_T1_* = −0.17, *t*(38) = 3.54, *p* = 0.001, *d* = 0.57. The endpoints in both summary conditions do appear to show a gradual return to the individual mean (Figure 5A). We compared postpenalty endpoints with a 2 × 3 × 5 ANOVA (Supplementary Table S3) with the between-participant factors *feedback modality* and *feedback schedule* and the within-participant factor *postpenalty trial* (T1 to T5). Postpenalty behavior differed depending on the feedback schedule, *F*(2, 109) = 14.05, *p* < 0.001, $\eta_{p}^{2}$ = 0.205, reflecting larger deviations from the individual mean for blocked summary feedback. Crucially, however, we found an interaction between postpenalty trial number and feedback schedule, *F*(8, 436) = 2.66, *p* = 0.007, $\eta_{p}^{2}$ = 0.046, suggesting that the postpenalty time courses (Figure 5A) are different for the different feedback schedules. Linear regressions fitted to the postpenalty trials of individuals showed a positive slope for blocked summary, *M_slope_* = 0.034, *t*(38) = 4.22, *p* < 0.001, *d* = 0.95, and for rolling summary, *M_slope_* = 0.029 *t*(38) = 2.13, *p* = 0.040, *d* = 0.48, but not for trial-by-trial feedback, *M_slope_* = −0.012, *t*(36) = −1.23, *p* = 0.227, *d* = −0.29. This reflects that endpoints immediately return to the individual mean with trial-by-trial feedback, whereas they show a more gradual return with summary feedback.

An immediate return to the individual mean (from T0 to T1) with a subsequent (T1 to T5) slope of (approximately) zero, as it is found with trial-by-trial feedback, does not necessarily reflect an active error correction process but may instead reflect a regression to the mean. Under summary feedback, the return to the individual mean after an error is incomplete and unfolds gradually over time. Is the gradual return the default pattern that can also be observed in the absence of any feedback? To test this, we repeated the same analysis for the blocked summary condition, this time only selecting trials during which no feedback was displayed between T0 and T5 (Supplementary Figure S4). Also in this selection, endpoints immediately following an error (T1), *M_T1_* = −0.34, were different from error trials (T0), *t*(38) = 7.86, *p* < 0.001, *d* = 1.49, as well as different from the individual mean, *t*(38) = 6.7, *p* < 0.001, *d* = 1.52. Most importantly, endpoints again showed a gradual return to the individual mean, *M_slope_* = 0.029, *t*(38) = 3.53, *p* = 0.001, *d* = 0.80. This shows that the gradual return that can be observed in the summary feedback groups (Figure 5A) does occur independent of feedback.

### Endpoint adjustment after blocked feedback

Is blocked summary feedback thus effective in guiding movement planning? To test whether information provided by blocked summary feedback was used, we compared prefeedback and postfeedback trials in the blocked summary condition (Figure 5B). Postfeedback endpoints were higher (i.e., more to the right) than those in prefeedback trials (main effect feedback phase), *F*(1, 38) = 9.58, *p* = 0.004, $\eta_{p}^{2}$ = 0.201 (Supplementary Table S4). This suggests that participants became more cautious after blocked summary feedback. When directly comparing trials before and after blocked feedback, this was especially evident after error-based feedback in the noisy block (veridical: *t*(19) = 2.09, *p* = 0.050, *d* = 0.47, noisy: *t*(19) = 3.74, *p* = 0.001, *d* = 0.83) but not with reinforcement feedback (veridical: *t*(19) = 0.17, *p* = 0.87, *d* = 0.04, noisy: *t*(19) = 0.91, *p* = 0.376, *d* = 0.20).

## Discussion

We tested the role of feedback for movement planning under risk. We asked participants to make saccades to an elongated white bar that consisted of a reward and a penalty region while manipulating the feedback modality (reinforcement vs. error-based) and the feedback schedule (trial-by-trial, blocked summary, rolling summary). The different schedules differed in terms of feedback frequency (blocked summary vs. rolling summary) or feedback focus (trial-by-trial vs. rolling summary). To perform well in this task, participants needed to select an aiming location that maximized their reward. The optimal aim point depended jointly on the reward landscape and on each participant's motor variability. While the reward structure was constant across participants (Figure 1B), the effective variability differed across individuals and conditions. In the veridical feedback block, participants could infer their own motor variability directly from feedback. In the second (noisy) block, we manipulated perceived variability by adding random perturbations to the feedback signals. As predicted, participants became more cautious when feedback implied higher variability. Together with the performance improvements observed at the start of the experiment (Supplementary Figure S3), this demonstrates that participants flexibly adapted their behavior to the task constraints. In terms of overall performance, results showed large variability between participants, spanning the whole spectrum of different strategies (risk-seeking, optimal, loss-aversive; Figures 2^3^–4). Trial-by-trial reinforcement feedback enabled a high level of performance for all participants. This was achieved by selecting an aim point (i.e., mean endpoint) that was either close to optimal or risk-seeking.

Our results therefore provide evidence that, within the present task context, trial-by-trial feedback is particularly effective when performing sensorimotor decisions under risk. Most importantly, our results suggest that feedback is most effective when provided on a trial-by-trial basis and when it simultaneously focuses on the outcome of a single trial compared to focusing on summary statistics of a group of trials. This latter aspect of feedback focus may appear counterintuitive, given that the decision where to aim depends on the variability of one's own movement—which can be more easily estimated from summary feedback. We had decided to provide summary feedback (mean, range, and quartiles) rather than displaying endpoints (error-based) or score points (reinforcement) of the recent 30 trials, because we reasoned that the latter would not allow a fair comparison between error-based and reinforcement feedback: Although multiple endpoints and their variability may be assessable at a glance in a scatterplot (error-based feedback), a display of 30 score points (reinforcement) may be overwhelming and may require a prolonged and more detailed processing to obtain the same information. We acknowledge, however, that the relatively abstract format of our summary feedback (i.e., summary statistics rather than visual distributions) may have contributed to the poorer performance observed in the summary groups, as it might have been less intuitive for participants. However, as has been shown by Ota et al. (2019), displaying a distribution of endpoints does not improve performance beyond that of trial-by-trial feedback. Moreover, we knew that our sample, which consisted exclusively of Psychology undergraduates, was well familiar with means, quartiles, and ranges (although these words were not used for the instructions; see Methods). Thus, we also cannot rule out the possibility that summary feedback is even less effective in a sample that is less familiar with statistics and distributions.

According to the guidance hypothesis (Salmoni et al., 1984; Schmidt et al., 1989), feedback with a reduced frequency can be more effective in motor learning and skill acquisition. However, the hypothesis mainly addresses performance in a retention test without any feedback—and not performance during the initial skill acquisition. Given that we did not include a retention test (i.e., a phase without any feedback), we cannot test this core prediction of the guidance hypothesis. Therefore, our data do not allow for a direct evaluation of this hypothesis. Nevertheless, in the specific context of movement planning under risk, we consider it less likely that reduced feedback frequency would provide an advantage. To perform well in the task at hand, one must be able to select an aiming location based on one's own motor uncertainty. Once participants have learned to choose a high amount of reward (as in the trial-by-trial group), there is little reason to expect a substantial decline in a later retention test. In contrast, since participants receiving summary feedback generally learned this mapping less effectively, it is unlikely that they would outperform the trial-by-trial group when feedback is removed. Hence, we would expect that the superiority of trial-by-trial reinforcement feedback for movement planning under risk would also persist in an immediate or delayed retention test.

Our findings show that trial-by-trial feedback is more successful when it provides information about the outcome of the behavioral goal (reinforcement) compared to information about the outcome of the movement (error-based). Although success or failure is immediately apparent with reinforcement feedback, translating feedback about one endpoint into success or failure requires an additional step. For example, for an endpoint close to the bar center, one needs to assess whether the displayed feedback was on the left-hand (penalty) or the right-hand half (reward). In that case, participants would have to perform a line bisection task, which is governed by visual uncertainty. In our task, visual uncertainty could have been reduced by providing a visual reference, either by highlighting the center of the bar or by having different-colored reward and penalty regions. Hence, we cannot rule out the possibility that participants in the error-based condition would have performed better when an additional visual reference had been provided. However, we believe it is unlikely that the presence or absence of a visual reference can account for all differences between the error-based and the reinforcement groups, considering that approximately one fourth of the trial-by-trial error-based group pursued a loss-aversive strategy.

Our results also show that people change their movement planning based on perturbed feedback to become more cautious. Yet, this adjustment was comparatively small and covered only approximately 25% of the adjustment expected, given the manipulation. One reason might be that participants attributed the larger errors in the second block externally, especially since this was emphasized in the instruction. Whereas oculomotor behavior can be adjusted to feedback indicating external errors (Heins & Lappe, 2024), this adjustment might have been incomplete (Gastrock, Modchalingam, ‘t Hart, & Henriques, 2020; Wilke, Synofzik, & Lindner, 2013). Alternatively, this incomplete adjustment might reflect the suboptimality in movement planning under risk (Ota et al., 2016; Wu et al., 2006).

Here, we established a region of optimality by computing both the upper and lower limits of optimality instead of relying on point estimates. Unlike some previous studies, we did not obtain a separate estimate of pure motor variability prior to the task. We deliberately refrained from doing so because motor variability is highly task-dependent and target-dependent and cannot be assessed in isolation for spatially extended targets like the horizontal bar used in our study. Without a meaningful reward structure, participants would have no fixed aim point, whereas instructing them to always look, for example, at the center of the bar would have changed saccade direction and amplitude with respect to the main experiment—which in turn affects endpoint variability (van Beers, 2007). In other words, endpoint variability in our task conflates both motor variability and aiming variability. By attributing all variability to the motor component (upper limit) or assuming a maximally variable aim point (and therefore minimal motor variability; lower limit), we have identified the possible span of the true motor variability. Because our approach is based on these boundary values (see Supplementary Figures S1, S2), it can be considered a conservative approach to estimate optimality. Hence, behavior outside that region can be clearly labeled suboptimal, as too risk-seeking or too cautious/loss-averse. Thus, the current approach considers both the task and the target, and it might therefore provide a more ecologically valid estimate of motor variability. Nevertheless, we acknowledge that directly measuring motor variability in a separate task could have provided complementary information about individual differences. Such estimates might generalize to some extent across similar tasks and targets, and future studies could benefit from including both direct and task-derived measures of variability. Importantly, however, the present conclusions are based on conservative boundary estimates and are therefore robust in the exact decomposition of motor and aiming variability.

In almost all groups, we observed both kinds of suboptimality (Figure 2C): risk-seeking and loss aversion. One notable exception was the group receiving trial-by-trial reinforcement feedback, in which the deviation from optimal was lowest (Figure 4C) and in which participants could be classified as optimal or risk-seeking. Thus, even in the condition resulting in the best performance, we see an overall tendency for suboptimality. One reason might be that people cannot represent asymmetric reward structures (Wu et al., 2006). In our task, the maximum penalty was twice as high as the maximum reward. Indeed, if the maximum penalty and reward had the same magnitude, the region of optimality would have been closer to the bar center (0.59°–1.01° for the trial-by-trial reinforcement group in the veridical condition), resulting in larger overlap between behavior and optimality. Alternatively, suboptimality might result from a need for certainty, and occasional penalties reassure participants that they are aiming at a location yielding a high reward rate. The latter would predict that participants can perform well under an asymmetric reward, yet they would rather do so when the maximum penalty is below the maximum reward—and not when it is the other way around—as in the current task.

With trial-by-trial feedback, endpoints immediately returned to the individual mean after a penalty (Figure 5A). This is consistent with a simple statistical assumption: If trial outcomes are sampled from a normal distribution around a fixed aim point, extreme values (e.g., penalties) will naturally be followed by less extreme values due to regression to the mean. In contrast, with summary feedback, the return to the mean is incomplete and unfolds gradually over several trials. Importantly, however, the magnitude of this gradual adjustment is very small (≈0.03° per trial) and therefore substantially smaller than typical saccadic endpoint variability. This suggests that, although statistically detectable, these temporal dependencies likely have limited functional relevance for the overall performance. Moreover, the small size of these adjustments makes it highly unlikely that they account for the substantial between-group differences in endpoints and reward rates observed in this study. One possible explanation for the postpenalty behavior under summary feedback is a slow drift in the participants’ internal aim point, driven by uncertainty about where exactly to aim. Without immediate feedback about an individual movement, participants may lose track of their intended target location, leading to a prolonged deviation across trials. Such drift would not only increase the overall endpoint variability (as observed in Figure 4A) but also delay the recovery from a penalty region. This phenomenon may be particularly pronounced in tasks with a spatially extended target and no additional visual reference. Under this interpretation, the immediate return to the mean with trial-by-trial feedback reflects a stabilization of the internal aim point: Participants can rapidly recalibrate their intended target location because each movement is followed by unambiguous feedback about the recent movements. Thus, while small posterror adjustments exist in all conditions, their effect is minimal, and trial-by-trial feedback appears to prevent slow drift from accumulating over time.

Our feedback manipulation was motivated by conceptual differences between sensory error–based and outcome-based learning processes. Sensory error–based mechanisms would predict direction-specific adjustments linked to the discrepancy between intended and observed movement outcomes, whereas outcome-based or reinforcement-like mechanisms would predict updates that depend on the success or failure of the chosen aim point, without encoding error direction. However, our experiment was not designed to distinguish these mechanisms, and the present behavioral data cannot differentiate between them. The observed advantages of trial-by-trial reinforcement feedback therefore reflect the behavioral consequences of providing outcome-focused information about individual movements, rather than evidence for a specific underlying learning algorithm. Future work combining our task with explicit computational modeling may help to clarify the mechanistic contributions of these learning processes.

Our findings were obtained using saccadic eye movements as a model of sensorimotor decision-making under risk. While saccades have specific properties, the underlying decision process—selecting an optimal movement endpoint under uncertainty—is shared across a wide range of effectors, including pointing movements. Thus, the mechanisms by which feedback guides the adjustment of movement planning are likely to generalize beyond the oculomotor domain, although future work is needed to confirm this. From an applied perspective, our results imply that training programs providing immediate, trial-by-trial reinforcement feedback may be particularly effective in contexts that require rapid mapping between actions and outcomes, such as sports training, surgical skill acquisition, or motor rehabilitation. Conversely, feedback formats that rely on statistical summaries may be less intuitive and therefore less effective in facilitating movement planning.

To summarize, we here show that trial-by-trial reinforcement feedback is superior when performing sensorimotor decisions under risk. Poorer performance in the other groups might be explained by additional visual uncertainty when translating endpoints into score values (error-based feedback) or by difficulties in translating the displayed summary feedback into an effective aiming strategy. Participants receiving trial-by-trial reinforcement feedback, in contrast, were most consistent across individuals, selecting an aim point yielding a high rate of reward. This pattern suggests that trial-by-trial reinforcement feedback represents the most intuitive and directly interpretable form of feedback, enabling participants to align their behavior efficiently. Crucially, trial-by-trial feedback reinforcement is effective because it conveys information about a single movement (feedback focus) and not because it is administered after every trial (feedback frequency).

## Supplementary Material

Supplement 1
