# Supplementary material for: The role of feedback for sensorimotor decisions under risk
Source: J Vis. 2026 Jan 22;26(1):13. doi: 10.1167/jov.26.1.13 (PMC12849826; doi:10.1167/jov.26.1.13)
Supplement: Supplement 1 [file jovi-26-1-13_s001.pdf]

Supplementary Material

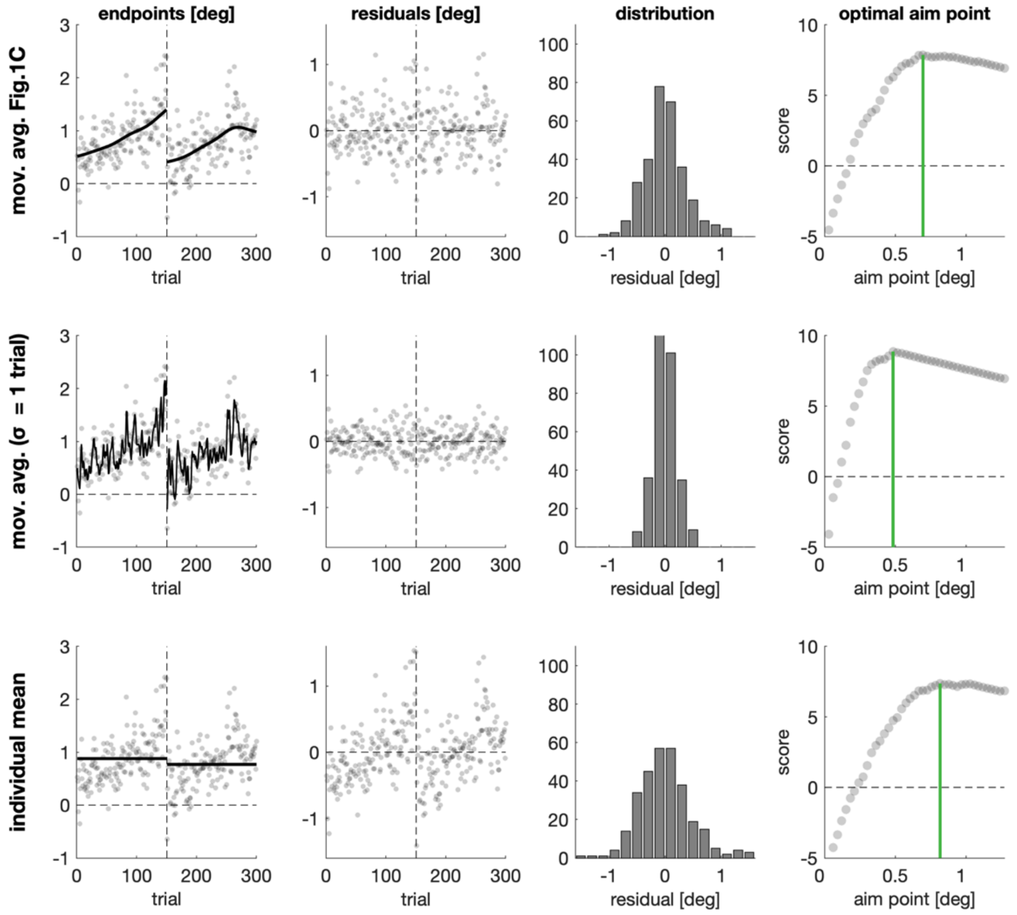

Suppl. Fig. S1. Region of optimality. Approach for computing the region of optimality based on the individual data shown in Figure 1C. Each row shows the computation of an optimal aim point (green line in right column). Either derived using the moving average depicted in Fig. 1C (top row), the moving average with the smallest sigma parameter (center row) or using the individual mean (bottom row). Whereas the moving average with the smallest sigma provides the lowest possible estimate for the optimal aim point, using the individual mean provides the highest estimate. The region of optimality spans the range between the lowest and the highest estimate (i.e. from the green line in the center row to the green line in the bottom row).

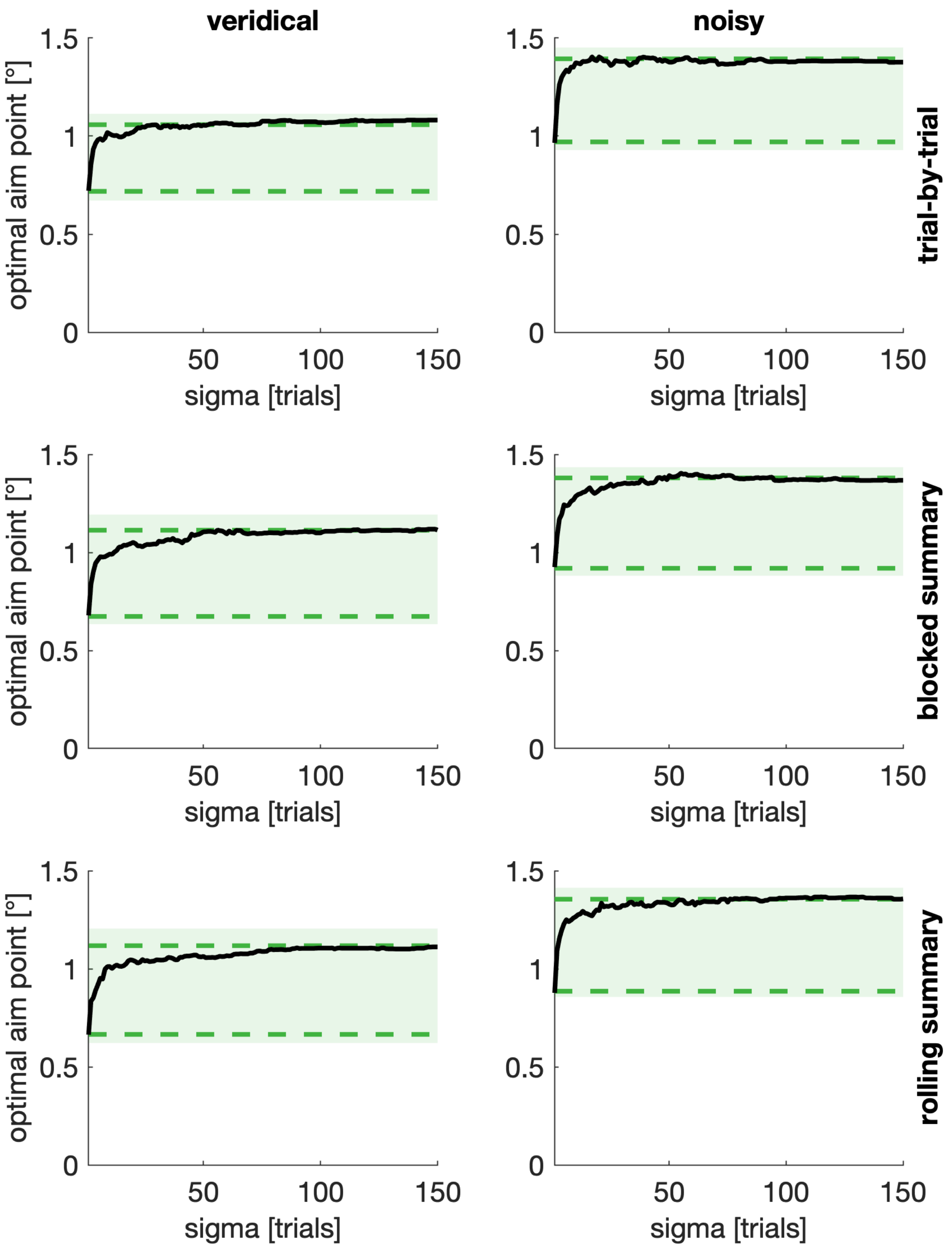

Suppl. Fig. S2. Sensitivity analysis examining the effect of varying the Gaussian window width (σ) on the estimated optimal aim point. Black lines show the estimated optimal aim point for σ values ranging from 1 to 150 trials. The green dashed lines indicate the lower bound (σ = 1 trial) and the upper bound (residuals computed relative to the blockwise mean, i.e., no Gaussian filtering). Shaded regions represent the resulting "region of optimality" as shown in Figure 2C, defined as the range between the lower limit minus 1.96 times the standard error and the upper limit plus 1.96 times the standard error.

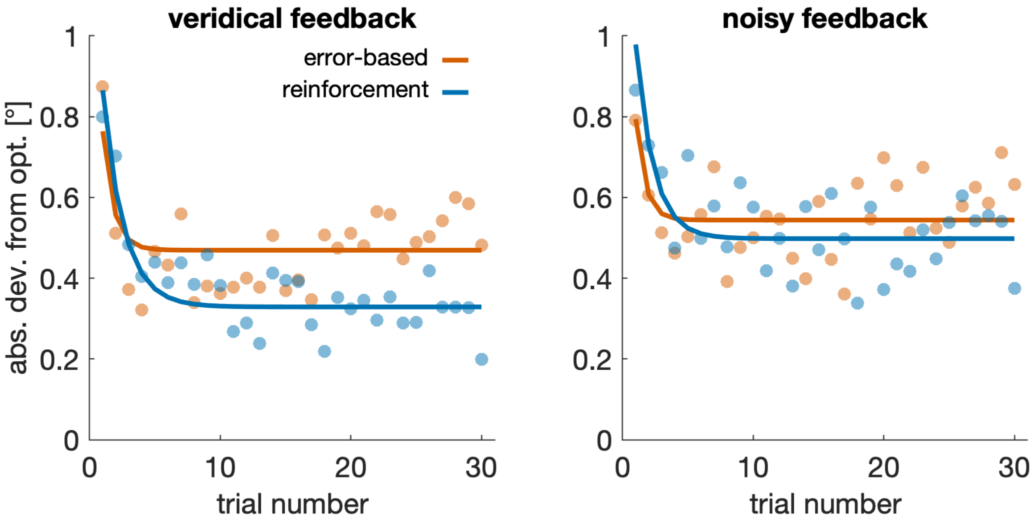

Suppl. Fig. S3. Early learning in the trial-by-trial feedback groups. Absolute deviation from each participant's region of optimality during the first 30 trials of the veridical block (left panel) and the noisy block (right panel). Blue and orange lines denote the reinforcement and error-based feedback groups, respectively. Each data point represents the mean value across participants for a given trial. Solid lines indicate the best-fitting exponential functions, illustrating a rapid reduction in deviation during the early trials, consistent with initial learning.

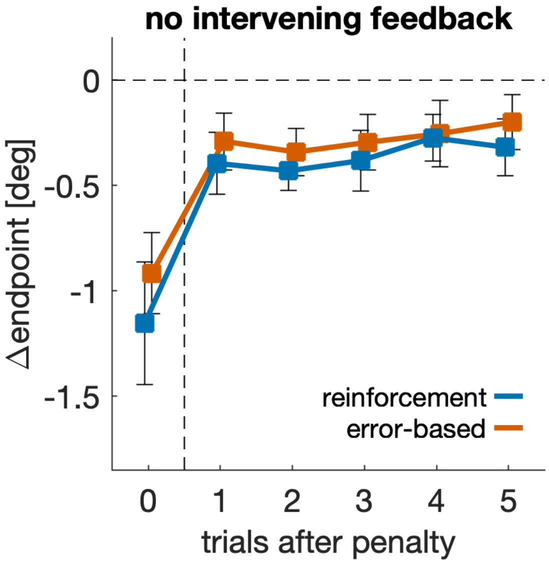

Suppl. Fig. S4. Post-penalty behavior without intervening feedback. Endpoints relative to the individual mean after encountering a penalty. Data from the blocked summary condition with no feedback in between the penalty trial (T0) and the first five trials preceding the penalty (T1 to T5). The figure/analysis is based on 999 penalties out of the 1163 penalties underlying the blocked summary panel in Figure 5A. Error bars are 95% confidence intervals of between-participant variability.

Supplementary Table S1

Results of a 2 × 2 × 3 ANOVA on the obtained scores (Fig. 2A) with the within-participant factor feedback veridicality (veridical, noisy) and the two between-participant factors feedback modality (error-based, reinforcement) and feedback schedule (trial-by-trial, blocked summary, rolling summary).

Variable	F	df	p	η_p^2
Veridicality	13.694	1,114	< .001	0.107
Modality	4.008	1,114	0.048	0.034
Schedule	2.615	2,114	0.078	0.044
Veridicality × Modality	0.015	1,114	0.902	< .001
Veridicality × Schedule	0.384	2,114	0.682	0.007
Modality × Schedule	4.628	2,114	0.012	0.075
Veridicality × Modality × Schedule	0.589	2,114	0.556	0.010

Supplementary Table S2

Results of a 2 × 2 × 3 ANOVA on the saccade endpoints (Fig. 2C) with the within-participant factor feedback veridicality (veridical, noisy) and the two between-participant factors feedback modality (error-based, reinforcement) and feedback schedule (trial-by-trial, blocked summary, rolling summary).

Variable	F	df	p	η_p^2
Veridicality	9.235	1,114	0.003	0.075
Modality	0.075	1,114	0.785	< .001
Schedule	0.533	2,114	0.588	0.009
Veridicality × Modality	0.236	1,114	0.628	0.002
Veridicality × Schedule	0.637	2,114	0.530	0.011
Modality × Schedule	4.697	2,114	0.011	0.076
Veridicality × Modality × Schedule	0.726	2,114	0.486	0.013

Supplementary Table S3

Results of a 2 × 3 × 5 ANOVA on the saccade endpoints with the between-participant factors feedback modality (error-based, reinforcement) and feedback schedule (trial-by-trial, blocked summary, rolling summary) and the within-participant factor post-penalty trial (T1 to T5).

Variable	F	df	p	η_p^2
Modality	0.061	1,109	0.806	< .001
Schedule	14.052	2,109	< .001	0.205
Post-penalty trial	5.146	4,436	< .001	0.045
Modality × Schedule	1.872	2,109	0.159	0.033
Modality × Post-penalty trial	0.551	4,109	0.698	0.005
Schedule × Post-penalty trial	2.657	8,436	0.007	0.046
Modality × Schedule × Post-penalty trial	0.674	8,436	0.715	0.012

Supplementary Table S4

Results of a 2 × 2 × 2 × 5 ANOVA on the saccade endpoints in the blocked summary condition, with the between-participant factor feedback modality (error-based, reinforcement) and the within-participant factors feedback veridicality (veridical, noisy), feedback phase (pre-feedback, post-feedback) and trial lag (T1 to T5; relative to feedback).

Variable	F	df	p	η_p^2
Veridicality	0.009	1,38	0.925	< .001
Modality	1.426	1,38	0.240	0.036
Phase	9.580	1,38	0.004	0.201
Lag	2.005	4,152	0.097	0.050
Veridicality × Modality	0.283	1,38	0.598	0.007
Veridicality × Phase	3.275	1,38	0.078	0.079
Veridicality × Lag	2.675	4,152	0.034	0.066
Modality × Phase	1.478	1,38	0.232	0.037
Modality × Lag	1.886	4,152	0.116	0.047
Phase × Lag	0.769	4,152	0.547	0.020
Veridicality × Modality × Phase	3.298	1,38	0.077	0.080
Veridicality × Modality × Lag	0.854	4,152	0.493	0.022
Veridicality × Phase × Lag	2.266	4,152	0.065	0.056
Modality × Phase × Lag	0.853	4,152	0.494	0.022
Veridicality × Modality × Phase × Lag	1.392	4,152	0.239	0.035

Supplementary Table S5. Endpoint classification.

Based on the individual region of optimality, endpoints were classified as penalty, risk-seeking (i.e., in between bar center and region of optimality), optimal or loss-aversive (i.e., beyond the region of optimality). Each cell shows the mean percentage of trials as well as the 95% confidence interval for a given condition.

condition			percentage of trials			
feedback type	feedback schedule	feedback veridicality	penalty	risk-seeking	optimal	loss-aversive
rein-forcement	trial-by-trial	veridical	M = 10.4% [7.2% 13.6%]	M = 46.7% [42.8% 50.6%]	M = 20.2% [16.9% 23.5%]	M = 22.7% [17.0% 28.4%]
		noisy	M = 12.3% [7.5% 17.0%]	M = 58.9% [53.8% 64.1%]	M = 15.8% [11.1% 20.4%]	M = 12.9% [9.0% 16.8%]
	blocked summary	veridical	M = 8.7% [3.2% 14.1%]	M = 26.2% [20.3% 32.1%]	M = 22.3% [17.6% 26.9%]	M = 42.8% [31.4% 54.1%]
		noisy	M = 4.7% [2.5% 6.9%]	M = 37.5% [28.5% 46.5%]	M = 22.2% [16.7% 27.8%]	M = 35.6% [23.7% 47.5%]
	rolling summary	veridical	M = 13.6% [8.1% 19.1%]	M = 33.0% [26.9% 39.1%]	M = 23.0% [18.3% 27.7%]	M = 30.3% [20.0% 40.5%]
		noisy	M = 10.9% [7.6% 14.1%]	M = 47.7% [38.6% 56.7%]	M = 18.9% [13.9% 23.8%]	M = 22.6% [12.1% 33.2%]
error-based	trial-by-trial	veridical	M = 10.5% [4.4% 16.6%]	M = 28.9% [20.9% 37.0%]	M = 20.8% [15.0% 26.6%]	M = 39.7% [26.4% 53.0%]
		noisy	M = 8.9% [4.3% 13.5%]	M = 39.0% [28.3% 49.7%]	M = 18.7% [14.1% 23.3%]	M = 33.4% [19.4% 47.4%]
	blocked summary	veridical	M = 16.8% [10.3% 23.3%]	M = 30.9% [24.0% 37.9%]	M = 20.5% [15.8% 25.1%]	M = 31.7% [20.4% 43.1%]
		noisy	M = 14.3% [7.1% 21.5%]	M = 40.5% [31.2% 50.0%]	M = 20.7% [15.3% 26.1%]	M = 24.4% [13.0% 35.9%]
	rolling summary	veridical	M = 11.1% [6.5% 15.7%]	M = 32.8% [24.5% 41.1%]	M = 21.0% [16.6% 25.5%]	M = 35.0% [22.4% 47.5%]
		noisy	M = 9.8% [4.3% 15.3%]	M = 43.2% [32.0% 54.4%]	M = 21.9% [16.8% 27.0%]	M = 25.1% [13.0% 37.2%]
